# Intrapericardial Extra-Anatomic Aorto-Aortic Bypass for Aortic Coarctation in Adults

**DOI:** 10.21470/1678-9741-2024-0185

**Published:** 2025-05-23

**Authors:** Enrique Seguel Soto, Gustavo Barril Merino, Aleck Stockins Larenas, Roberto González Lagos, Rodrigo Reyes Melo

**Affiliations:** 1 Department of Surgery, Faculty of Medicine, University of Concepción, Concepción, Chile; 2 Cardiovascular Center, Guillermo Grant Benavente Hospital of Concepción, Concepción, Chile; 3 General Surgery Resident, Pontifical Catholic University of Chile Program, Santiago, Chile

**Keywords:** Coronary Artery Disease, Aortic Valve, Polyethylene Terephthalates, Aortic Coarctation, Survival Rate, Follow-Up Studies, Aortic Valve Stenosis, Aortic Valve Disease, Prostheses and Implants, Coronary Artery Bypass

## Abstract

**Introduction:**

The preferred treatment for aortic coarctation is direct repair during childhood. However, some patients reach adulthood without being diagnosed. For these patients, an extra-anatomic bypass offers an alternative solution.

**Objective:**

To evaluate the surgical outcomes of adult patients with aortic coarctation treated with an extra-anatomic aorto-aortic bypass.

**Methods:**

This retrospective study includes adult patients who underwent an intrapericardial extra-anatomic bypass using a Dacron® tube from 2013 to 2021 (n=8). Clinical characteristics, surgical outcomes, survival rates, and the need for reinterventions were assessed up to March 31, 2024.

**Results:**

All patients were male, with an average age of 39.9 ± 10.8 years (range 23-51). All were hypertensive. Four patients had associated aortic valve disease, and one had coronary artery disease. The operative risk, calculated using the European System for Cardiac Operative Risk Evaluation II score, was 1.65%. Four patients underwent concurrent valve surgeries (two valve replacements, one David procedure, and one Bentall procedure), and one had coronary artery surgery. The average pump time was 119 minutes, with longer times for those undergoing additional procedures (157 vs. 82.5 minutes). There was no operative mortality. The mean follow-up period was 107.1 ± 32 months, during which all patients survived. One patient required reintervention on the 118^th^ postoperative month due to aortic stenosis, necessitating valve replacement with a biological prosthesis.

**Conclusion:**

Intrapericardial extra-anatomic bypass is a viable option for treating aortic coarctation in adults, demonstrating excellent shortand long-term outcomes. It can be effectively combined with other surgical procedures.

## INTRODUCTION

**Table:** 

Abbreviations, Acronyms & Symbols	
CPB	= Cardiopulmonary bypass
CT	= Computed tomography
EuroSCORE	= European System for Cardiac Operative Risk Evaluation

Aortic coarctation is a congenital heart defect characterized by the narrowing of the aorta at the isthmus, accounting for 5-7% of congenital heart diseases and affecting three per 10,000 live births. This defect varies from localized stenosis to hypoplasia of the aorta and can be associated with other anomalies like bicuspid aortic valve, ventricular septal defect, patent ductus arteriosus, and more. Symptoms vary based on the degree of coarctation and associated lesions^[1]^.

In 2008, The American College of Cardiology and American Heart Association (or ACC/AHA) guidelines for adults with congenital heart disease recommended intervention for coarctation in the following settings: peak-to-peak coarctation gradient 20 mg, which is the difference in peak pressure proximal and beyond the narrowed segment, with imaging evidence of significant coarctation and radiologic evidence of significant collateral flow. The resting gradient alone may be an unreliable indicator of severity when there is significant collateral circulation^[2]^.

The first surgical repair was performed by Dr. Crafoord in 1944^[3]^. Currently, direct repair is the treatment of choice, typically performed in childhood via left thoracotomy. The procedure involves dissecting the aorta, resecting the stenotic segment, and performing end-to-end anastomosis, yielding excellent long-term results^[1^]. Other surgical options include patch aortoplasty, subclavian flap aortoplasty, and prosthetic tube interposition for older patients^[2^,^4^,^5]^.

Despite these advancements, some patients reach adulthood undiagnosed, developing hypertension and other complications^[6]^. Additionally, 5-30% of patients repaired in childhood may experience recurrence, requiring further intervention^[7]^. Treatment options for these cases include endovascular stenting and extra-anatomic bypasses like axillo-bifemoral or aorto-aortic bypass^[8^-^10]^.

The intrapericardial extra-anatomic bypass between the ascending and descending aorta via median sternotomy was first described by Vijayanagar in 1980, demonstrating reproducibility and safety with excellent midand long-term outcomes^[11]^. This technique was initiated in our hospital in 2013.

The aim of this study is to detail the surgical technique and to describe both the immediate and long-term outcomes in patients treated with this method.

## METHODS

### Patients

This retrospective study involves eight patients who underwent an intrapericardial extra-anatomic aorto-aortic bypass for aortic coarctation at Guillermo Grant Benavente Hospital (Concepción, Chile) between 2013 and 2021. Data were collected from the cardiovascular center’s cardiac surgery registry, and an anonymized database was constructed.

### Surgical Technique

Preoperative evaluation includes standard protocols based on the patient’s condition, incorporating thoracoabdominal and pelvic computed tomography (CT) angiography to identify associated congenital malformations (*e.g.*, lusory artery, bovine trunk), aneurysms, or atherosclerotic disease. This imaging also helps measuring the descending aorta’s diameter to determine the appropriate prosthesis size.

Patients undergo surgery under general anesthesia with standard cardiac surgery monitoring and cardiopulmonary bypass (CPB). Preparation includes draping both lower limbs for potential femoral vessel access if needed.

**Fig. 1:**
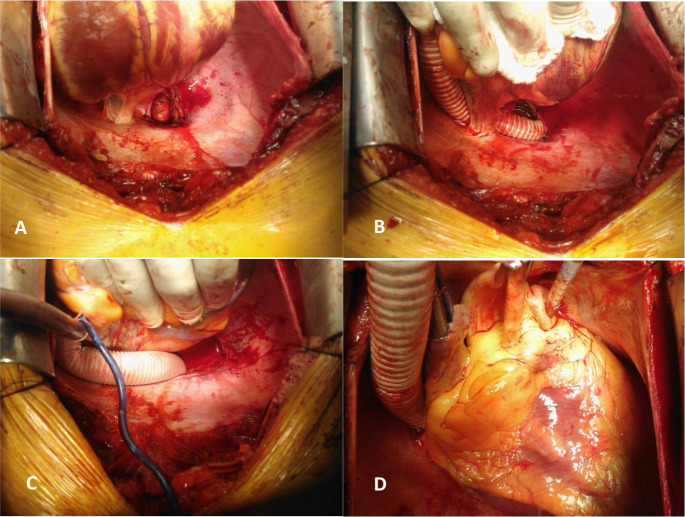
a) Dissection of the posterior pericardium and exposure of the descending aorta; b) passage of the prosthetic tube behind the inferior vena cava; c) passage of the tube in front of the vena cava; d) pressurization of the tube and measurement of its length.

A median sternotomy is performed, followed by opening the pericardium, full heparinization, placement of purse-string sutures, and connection to CPB with standard flows and temperatures. The heart remains beating while being displaced to expose the posterior pericardium. The descending aorta, typically small in diameter, is palpated and identified. The pericardium is opened along the midline, exposing the descending aorta above the diaphragm (Figure 1A).

A lateral clamp is applied to the aorta after reducing arterial pressure by lowering CPB flow to ensure proper clamping. A 15 mm longitudinal arteriotomy is made, followed by an end-to-side anastomosis with a 16, 18, or 20 mm Dacron® graft, depending on the aortic diameter, using continuous 5-0 polypropylene sutures. After completing the anastomosis, the clamp is removed, air is purged from the graft, and hemostasis is checked.

**Fig. 2:**
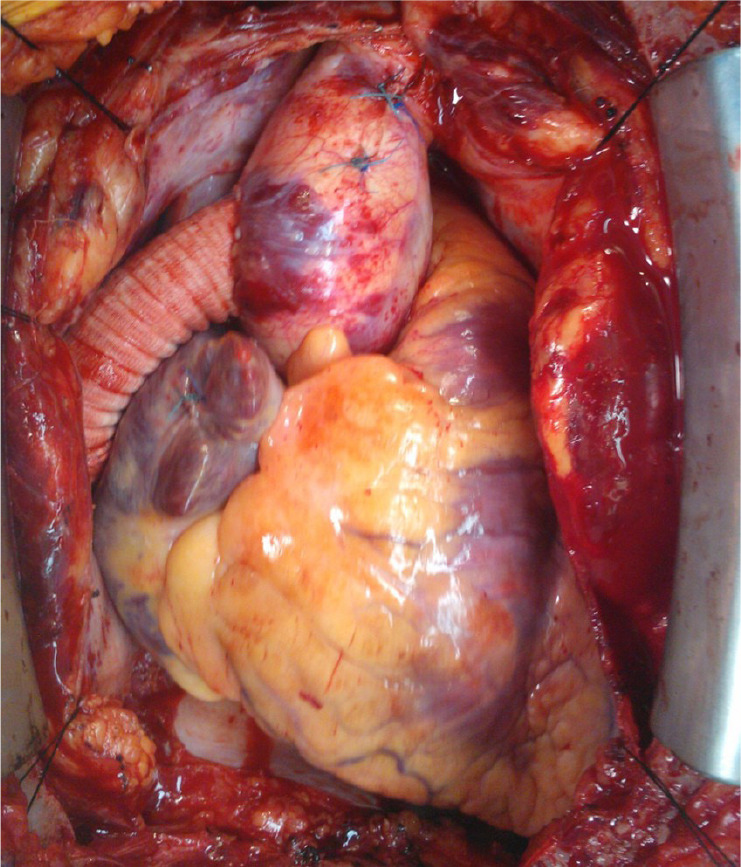
Completed aorto-aortic bypass.

The graft is passed anteriorly through the pericardial fold behind (Figure 1B) or in front of the inferior vena cava (Figure 1C). The graft length is measured to avoid kinking or traction on the anastomoses, keeping the graft pressurized with blood (Figure 1D). The ascending aorta is laterally clamped, and a longitudinal aortotomy is made for the proximal anastomosis using continuous 5-0 polypropylene sutures. Air is purged, and the clamp is removed. The surgery concludes with weaning from CPB, decannulation, and reversal of heparin with protamine. The result is shown in Figure 2. The pericardium is left open to prevent compression or kinking of the bypass.

### Variables

Demographic (sex, age), clinical, echocardiographic, and angiographic characteristics were studied. The operative risk was calculated using the European System for Cardiac Operative Risk Evaluation (EuroSCORE) II. Technical feasibility, associated surgeries, and CPB times were analyzed. Surgical complications (reoperation for bleeding, mediastinitis) and medical complications (cardiovascular, neurological, renal, pulmonary, infections), as well as operative mortality, were recorded.

### Follow-up

Clinical events such as long-term mortality, need for reinterventions, and cerebrovascular accidents were evaluated up to March 31, 2024.

## RESULTS

### Patients

All patients were male, with an average age of 39.9 ± 10.8 years (range 23-51 years). All of them had hypertension. Four patients had aortic valve pathology (two with stenosis and two with insufficiency associated with root dilation). One patient had associated coronary artery disease. The operative risk, calculated using EuroSCORE II, was 1.65 ± 0.32%.

### Surgeries

All procedures were completed using the described technique. Additionally, two patients underwent valve replacements with mechanical prostheses, one underwent Bentall procedure (replacement of the aortic valve with a mechanical prosthesis, root replacement with a Dacron® tube, and reimplantation of the coronary ostia), and one underwent David operation (root replacement with a Dacron® tube, reimplantation of the aortic valve, and reimplantation of the coronary ostia).

The average CPB time was 119 ± 57.7 minutes. For patients without associated surgeries, the CPB duration was 82.5 ± 8.7 minutes, and for those with additional surgeries, it was 157 ± 71 minutes.

### Complications and Operative Mortality

One patient who underwent the Bentall procedure experienced an issue with the left coronary ostium anastomosis, necessitating a coronary bypass to the left anterior descending and marginal arteries using a saphenous vein graft due to extensive development of the mammary artery and intercostal arteries. The patient showed clinical and electrocardiographic signs of ischemia, leading to a follow-up coronary angiography, which revealed a kinked graft that was corrected with a stent.

There were no infectious, renal, neurological, or other cardiovascular complications and no prolonged mechanical ventilation. No reoperation for bleeding or infection was required. There was no operative mortality.

The average intensive care unit stay was 4.2 days, and the average hospital discharge was 6.6 days.

### Follow-up

The average follow-up period was 107.1 ± 32 months (range 35-128 months).

**Fig. 3:**
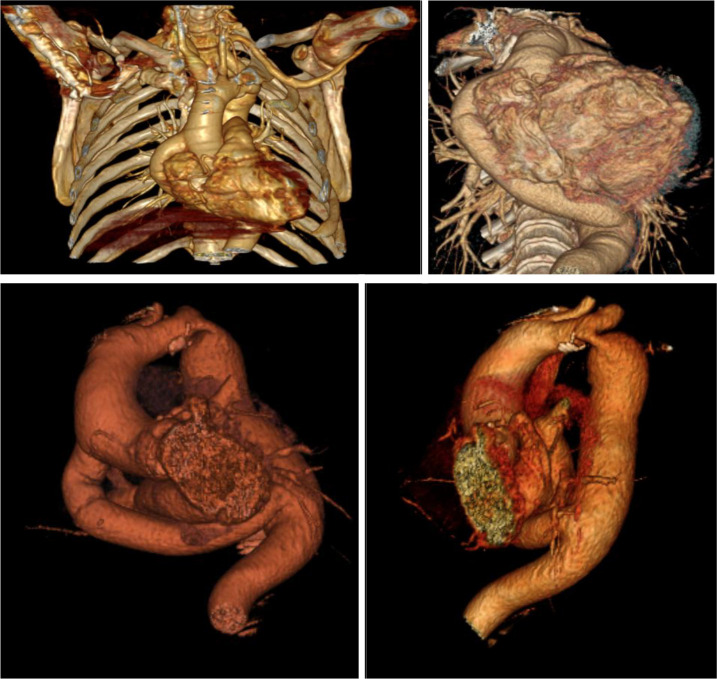
Reconstruction of an angio-computed tomography scan of a patient with the aorto-aortic tube (Vitrae®, zoom 143%; W/L: 236/294; VR: Base color).

One patient required reoperation on the 118^th^ postoperative month due to symptomatic severe aortic stenosis. A follow-up CT angiogram showed the prosthetic tube and an increase in the diameter of the descending aorta compared to its pre-implant size (Figure 3). An aortic valve replacement with a mechanical prosthesis was performed.

There were no deaths, endocarditis, or cerebrovascular accidents during the follow-up period. Clinically, all patients were in New York Heart Association functional class I.

## DISCUSSION

The construction of an extra-anatomic bypass to address pathologies of the distal aortic arch and proximal descending aorta is an alternative that resolves hemodynamic and distal perfusion issues without directly addressing the lesion. This is particularly advantageous for patients in whom direct approach is difficult, such as those with previously operated aortic coarctations, patients with prior thoracic surgery, or those with associated pathologies requiring heart involvement, serving as an option for centers lacking endovascular stenosis resolution capabilities^[12^,^13]^.

The first case utilizing this technique (not included in this series) involved a patient with hydatid disease affecting the descending thoracic aorta. An extra-anatomic bypass was performed to isolate the aorta, ligating it distally at the arch and proximally above the bypass, aiming for vascular control before a second surgical phase to remove the thoracic cyst. Immediate results were excellent, but surgery for hydatidosis was precluded by the emergence of central neurological compromise, leading to the patient's demise from cerebral hemorrhage at four years after operation.

Following this case, the technique was applied to the other patients described in this series. It is particularly indicated for patients requiring complementary procedures, such as aortic valve or ascending aorta surgery, necessitating sternotomy and CPB^[13^,^14]^. In two cases, aortic valve replacement was associated, and in two others, root surgery (one Bentall procedure and one David operation) was associated. However, as described by Dr. Schoenhoff et al., in isolated surgery, the technique can be performed without CPB^[15]^.

One patient who underwent a Bentall operation experienced issues with a coronary button, which was corrected via bypass. No fatalities or other complications occurred, and hospital stays were short, as expected for a series of young, low-risk patients^[16]^.

One patient developed aortic stenosis requiring surgery nine years after the initial intervention. Imaging studies showed prosthetic tube configuration and permeability, as well as increased descending aorta diameter compared to preoperative measurements.

In summary, the extra-anatomic bypass is a valuable surgical approach for managing complex aortic pathologies. Its implementation is associated with low morbidity and mortality rates, enabling the correction of hemodynamic complications arising from coarctation in a single surgical intervention. This technique holds value specially for patients burdened with multiple comorbidities or requiring additional surgical interventions^[17^,^18]^.

### Limitations

This is a small series of selected patients operated on over a period of eight years in a single centre. The results are influenced by selection bias and the experience of the surgical team. The nine-year follow-up is short, and a longer period of time must be expected to see if these results are maintained over time. There are alternative techniques that must be considered on a case-by-case basis before indicating this type of surgery.

## CONCLUSION

The construction of an aorto-aortic extra-anatomic bypass is a reproducible technique that allows correction of aortic coarctation in patients where direct approach is challenging, as well as other pathologies involving the isthmus or proximal descending aorta.
